# Solitary Subcutaneous Nodular Lymphoid Lesions in Dogs: Histopathologic and Immunophenotypic Comparison of B-Cell Pseudolymphoma and Subcutaneous Panniculitis-like T-Cell Lymphoma

**DOI:** 10.3390/vetsci12060532

**Published:** 2025-05-30

**Authors:** Young-Hyun Koo, Hyo-Sung Kim, Woo-Jin Kim, Hye-Ji Oh, Byoung-Je Lee, Chang-Kyun Im, Sun-Hee Do

**Affiliations:** 1 Department of Veterinary Clinical Pathology, College of Veterinary Medicine, Konkuk University, Seoul 05029, Republic of Korea; k12852@konkuk.ac.kr (Y.-H.K.);; 2 Deok-Chun Animal Hospital, 119, Mandeok-daero, Buk-gu, Busan 46554, Republic of Korea; 3 A-ji Animal Hospital, 289, Dokseodang-ro, Seongdong-gu, Seoul 04725, Republic of Korea

**Keywords:** cutaneous pseudolymphoma, subcutaneous panniculitis-like T-cell lymphoma, B-cell hyperplasia, T-cell lymphoma, lymhocyte-rich panniculitis

## Abstract

This report describes two cases of solitary subcutaneous nodular lymphoid lesions in dogs. Case 1 involved a 6-year-old male Maltese and Case 2 a 5-year-old female Yorkshire Terrier. Both presented with firm, non-ulcerated dorsal subcutaneous nodules and were unresponsive to corticosteroids. Surgical excision was performed for diagnosis. Histopathology and immunohistochemistry revealed distinct patterns. Case 1 exhibited well-formed lymphoid follicles with CD20^+^/PAX5^+^ B cells and strong BCL6 but absent BCL2 expression, consistent with B-cell pseudolymphoma. Case 2 demonstrated diffuse CD3^+^ T-cell infiltrates with adipocyte rimming and minimal BCL2/BCL6 expression, diagnostic for SPTCL. Despite their similar clinical presentation, these two lesions were histopathologically and immunophenotypically distinct. These findings underscore the importance of histologic and immunophenotypic correlation in accurately distinguishing benign from malignant subcutaneous lymphoid proliferations in dogs.

## Simple Summary

Canine cutaneous lymphoproliferative disorders range from reactive hyperplasia to malignant lymphomas and can be challenging to differentiate without immunophenotypic characterization. Subcutaneous panniculitis-like T-cell lymphoma (SPTCL) and pseudolymphoma may present with similar clinical features but require distinct clinical management. This report describes two dogs presenting with subcutaneous nodules and compares histopathological and immunohistochemical findings between SPTCL and pseudolymphoma. The findings emphasize the critical role of histological and immunophenotypic analyses in differentiating benign from malignant subcutaneous lesions, enabling appropriate clinical management in canine patients.

## 1. Introduction

Canine cutaneous lymphoproliferative disorders encompass a broad spectrum ranging from benign reactive infiltrates to aggressive lymphomas [1]. Subcutaneous panniculitis-like T-cell lymphoma (SPTCL) is a rare form of non-epitheliotrophic lymphoma that typically presents as firm subcutaneous nodules and exhibits a histologic pattern of adipocyte “rimming” by atypical T lymphocytes [2,3].

In contrast, cutaneous pseudolymphomas—also referred to as cutaneous lymphoid hyperplasia—are benign polyclonal infiltrates composed of well-differentiated T or B lymphocytes [2]. These lesions commonly manifest as dominant nodules and closely mimic lymphoma both clinically and histologically [3]. Accurate differentiation relies heavily on immunophenotyping and histologic characteristics, such as the presence of mixed T- and B-cell infiltrates and reactive germinal centers [4].

Due to their similar histological presentations, distinguishing pseudolymphomas from malignant lymphomas could be diagnostically challenging [5]. Misclassification may result in unnecessary chemotherapy in benign cases or delayed treatment in malignant ones. In particular, SPTCL often mimics inflammatory panniculitis or reactive processes in its early stages, increasing the likelihood of under- or misdiagnosis [6]. However, in veterinary medicine, to the author’s knowledge, studies are limited, and established diagnostic guidelines are lacking. This complicates clinical decision-making for solitary subcutaneous nodular lesions in dogs.

This case report describes and compares two solitary subcutaneous lymphoid lesions in dogs—one representing a benign B-cell pseudolymphoma and the other an instance of SPTCL—highlighting the need for careful diagnostic evaluation of subcutaneous lymphoid lesions and the use of immunohistochemistry (IHC).

## 2. Materials and Methods

### 2.1. Case Selection

Two middle-aged dogs were evaluated to have firm, non-ulcerated, subcutaneous nodules located in the dorsal region. Case 1 involved a 5-year-and-9-month-old spayed female Yorkshire Terrier; Case 2 involved a 6-year-old neutered male Maltese. Both dogs were unresponsive to systemic corticosteroid therapy (prednisolone administered once daily prior to biopsy: 0.5 mg/kg for approximately 7 days in Case 1, and 1 mg/kg for approximately 10 days in Case 2), and surgical excision was performed for definitive diagnosis.

Neither dog had a history of injection or vaccination at the sites where the subcutaneous nodules developed, suggesting that a vaccine-associated etiology was unlikely.

### 2.2. Histopathology and Immunohistochemistry

Tissues were formalin-fixed, paraffin-embedded, and sectioned for hematoxylin and eosin (H&E) staining. Immunohistochemistry was performed using the following primary antibodies: CD20 (Thermo-Fisher Scientific, Fremont, CA, USA), PAX5 (Invitrogen, Carlsbad, CA, USA), CD3 (Abcam, Cambridge, UK), BCL2 (Santa Cruz Biotech, Dallas, TX, USA), and BCL6 (Abcam, Cambridge, UK). Negative controls were prepared by omitting the primary antibody and incubating tissue sections with PBS or blocking buffer alone, to confirm the specificity of immunostaining. Antigen retrieval was conducted in citrate buffer at ~100 °C for 30 min using a pressure cooker. Detection was performed using the ImmPRESS^®^ HRP Horse Anti-Mouse and Anti-Rabbit IgG Polymer (Vector Laboratories, Newark, CA, USA). The target protein was visualized as a brown chromogen using the ImmPACT^®^ DAB Substrate Kit (Vector Laboratories, Newark, CA, USA); this was followed by counterstaining with Mayer’s Hematoxylin (ScyTek Laboratories, Logan, UT, USA).

## 3. Results

**Figure 1:**
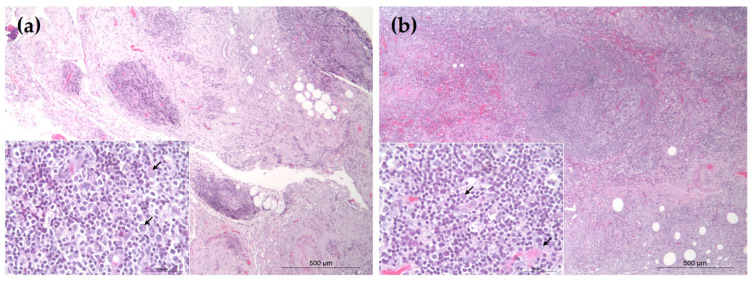
Histologic findings of subcutaneous lymphoid lesions. Hematoxylin and eosin (H&E) staining: (**a**) Case 1: the nodules are composed of mixed infiltrates of lymphocytes, histiocytes, and macrophages; (**b**) Case 2: the nodules predominantly consist of neoplastic lymphocytes with occasional histiocytes/macrophages and plasma cells. Scale bars = 500 μm and 50 μm (inset).

Histological evaluation of both cases revealed dense infiltrates of small- to intermediate-sized lymphocytes (nuclear size: 1.0–1.5 × RBC) with mild anisokaryosis. These cells infiltrated the subcutis in either lobular or diffuse patterns (Figure 1). Occasional infiltration of histiocytes, macrophages, and plasma cells was also observed (Figure 1, inset). Based on these features, differential diagnoses included B-cell pseudolymphoma and panniculitis-like B-cell lymphoma.

**Figure 2:**
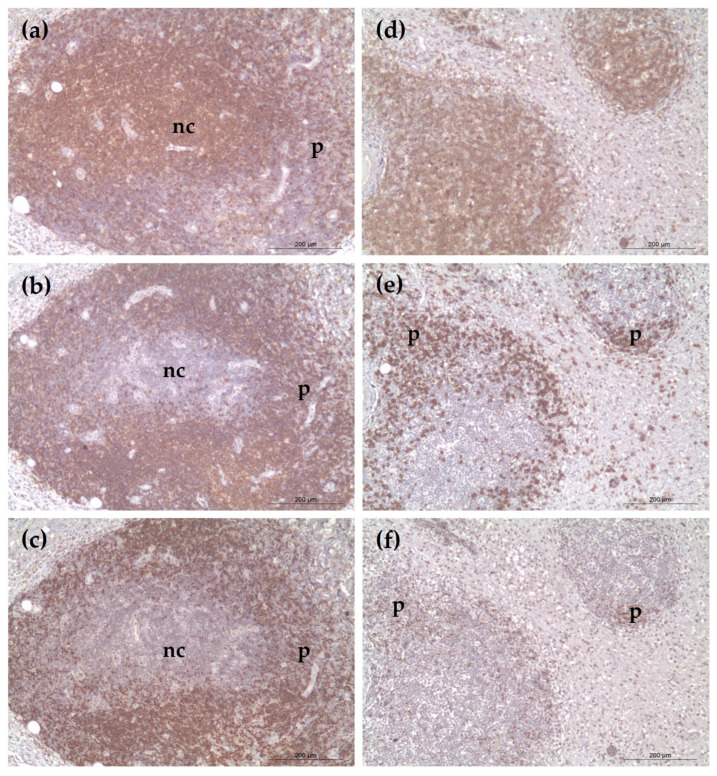
Immunohistochemical staining of neoplastic cells: (**a**–**c**) Case 1: the nodular periphery (p) shows strong positivity for CD20 (**b**) and PAX5 (**c**), with a few CD3-positive (**a**) lymphocytes observed within the center of the nodules (nc); (**d**–**f**) Case 2: CD3^+^ T-cell infiltration is evident (**d**), with scattered CD20^+^ (**e**) and PAX5^+^ (**f**) lymphocytes at the periphery (p). Scale bars = 200 μm.

**Figure 3:**
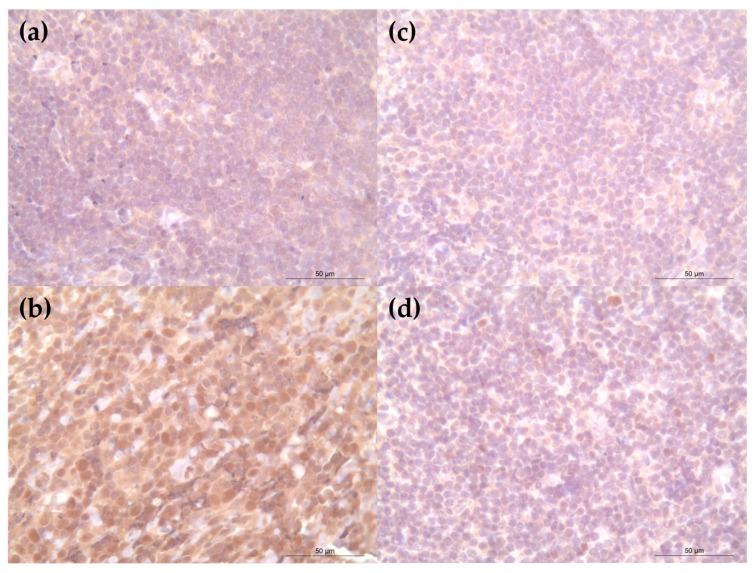
Expression of BCL2 and BCL6 expression in lymphoid cells: (**a**,**b**) Case 1: the nodular center is negative for BCL2 (**a**) but shows strong BCL6 expression (**b**); (**c**,**d**) Case 2: both BCL2 (**c**) and BCL6 (**d**) are negative in the infiltrating lymphocytes located at the nodular center. Scale bars = 50 μm.

Case 1 showed nodular clusters of CD20^+^ and PAX5^+^ B cells (Figure 2b,c). BCL2 was largely negative (Figure 3a), while BCL6 expression was confined to germinal center-like regions (Figure 3b)—findings supportive of reactive follicular hyperplasia.

Case 2 demonstrated a diffuse subcutaneous infiltrate of CD3^+^ T lymphocytes (Figure 2d) with minimal B-cell presence (Figure 2e,f). BCL2 and BCL6 expressions were weak or absent (Figure 3c,d). The pattern of lymphocyte infiltration and characteristic rimming of adipocytes was consistent with SPTCL (negative control slides showed no non-specific staining and were excluded from the figures).

## 4. Discussion

Canine cutaneous pseudolymphomas are rare lesions and can be easily misdiagnosed as malignant lymphomas due to their similar clinical presentation [5]. These benign lesions typically display well-formed lymphoid follicles with germinal centers and a mixed T- and B-cell population [1,5]. The immunophenotypic profile-predominance of CD20^+^ and PAX 5^+^ B cells with BCL6 expression and BCL2 negativity is characteristic of reactive nodal or cutaneous lymphoid hyperplasia, as opposed to most B-cell lymphomas [7,8].

Aberrant BCL2 expression is frequently seen in follicular lymphoma, whereas its absence in reactive follicles serves as an important discriminating marker [7,9]. While BCL-2-negative follicular lymphoma has been reported in cats [10], reports in dogs remain limited. Nevertheless, pseudolymphomas should be monitored over time and clonality testing considered, as rare cases may undergo malignant transformation [11].

Conversely, subcutaneous panniculitis-like T-cell lymphoma (SPTCL) is a rare and distinct neoplastic entity in dogs that warrants clinical recognition [12]. Case 2 exhibited classic histological and immunophenotypic features of SPTCL, including CD3^+^ T-cell infiltration, adipocyte rimming, and the absence of BCL2 and BCL6 expression [12]. These features highlight the importance of IHC in differentiating SPTCL from inflammatory panniculitides.

Alongside SPTCL, other lymphocyte-rich panniculitic conditions—such as lupus profundus (lupus panniculitis) and lymphocyte-rich cytotoxic T-cell panniculitis secondary to infection—must also be considered in the differential diagnosis [13,14].

However, these entities generally display additional histological or clinical features that allow for accurate distinction. Despite this, overlapping characteristics in early lesions may complicate diagnosis [15], underscoring the need for careful interpretation and, in some cases, adjunctive molecular testing.

Given the potential aggressiveness of SPTCL [12], early and accurate diagnosis is essential. Misclassification of these lesions has important therapeutic consequences. Overdiagnosis of lymphoma in a benign pseudolymphoma case could result in unnecessary chemotherapy, while underdiagnosing SPTCL may delay appropriate treatment. Therefore, a thorough histopathologic evaluation combined with IHC is critical. Although immunohistochemistry is a valuable tool in the classification of lymphoid infiltrates, it does not assess clonal composition. In diagnostically ambiguous cases, molecular clonality testing such as PCR for antigen receptor rearrangement (PARR) is essential for confirming the neoplastic nature of the lesion [16]. In the present cases, clonality testing was not performed, representing a diagnostic limitation. Nevertheless, the combined morphologic and immunophenotypic features were sufficient to support the differentiation between B-cell pseudolymphoma and SPTCL [17,18].

Follow-up was limited to twelve months post-surgery, during which no recurrence was observed. However, longer-term monitoring is necessary for evaluating the risk of recurrence or malignant transformation. Currently, there are no established treatment guidelines for canine pseudolymphoma or SPTCL.

From a clinical standpoint, pseudolymphoma should remain a differential diagnosis in all solitary, lymphocyte-rich, well-differentiated skin lesions, especially in otherwise healthy animals. At the same time, increased awareness of non-epitheliotropic T-cell lymphomas, such as SPTCL, is necessary as they may initially resemble inflammatory panniculitis and be misinterpreted as benign. Preventive strategies, appropriate biopsy sampling, and multidisciplinary diagnostic collaboration are essential for avoiding misdiagnosis and ensuring optimal patient care.

## 5. Conclusions

These two canine cases, presenting with clinically similar solitary subcutaneous nodules, illustrate the importance of a thorough diagnostic work-up. Although indistinguishable in gross appearance, histopathologic and immunophenotypic analyses revealed fundamentally different diseases processes—one reactive and benign, the other malignant and aggressive. Morphologic and immunophenotypic evaluation provided important diagnostic guidance in these cases; however, molecular clonality testing (e.g., PARR) would further substantiate the distinction between reactive and neoplastic lymphoid infiltrates, especially in diagnostically ambiguous cases.

## Data Availability

Not applicable. The raw data supporting the conclusions of this article will be made available by the authors on reasonable request.
